# Scale up, optimization and stability analysis of Curcumin C3 complex-loaded nanoparticles for cancer therapy

**DOI:** 10.1186/1477-3155-10-38

**Published:** 2012-08-31

**Authors:** Amalendu P Ranjan, Anindita Mukerjee, Lawrence Helson, Jamboor K Vishwanatha

**Affiliations:** 1 Department of Molecular Biology & Immunology and Institute for Cancer Research, Graduate School of Biomedical Sciences, University of North Texas Health Science Center, Fort Worth, TX, 76107, USA; 2 SignPath Pharmaceuticals Inc., Quakertown, PA, USA

**Keywords:** Scale up, Optimization, PLGA nanoparticles, Cancer, Response surface methodology (RSM), Curcumin C3 complex, Central composite design (CCD)

## Abstract

**Background:**

Nanoparticle based delivery of anticancer drugs have been widely investigated. However, a very important process for Research & Development in any pharmaceutical industry is scaling nanoparticle formulation techniques so as to produce large batches for preclinical and clinical trials. This process is not only critical but also difficult as it involves various formulation parameters to be modulated all in the same process.

**Methods:**

In our present study, we formulated curcumin loaded poly (lactic acid-co-glycolic acid) nanoparticles (PLGA-CURC). This improved the bioavailability of curcumin, a potent natural anticancer drug, making it suitable for cancer therapy. Post formulation, we optimized our process by Reponse Surface Methodology (RSM) using Central Composite Design (CCD) and scaled up the formulation process in four stages with final scale-up process yielding 5 g of curcumin loaded nanoparticles within the laboratory setup. The nanoparticles formed after scale-up process were characterized for particle size, drug loading and encapsulation efficiency, surface morphology, *in vitro* release kinetics and pharmacokinetics. Stability analysis and gamma sterilization were also carried out.

**Results:**

Results revealed that that process scale-up is being mastered for elaboration to 5 g level. The mean nanoparticle size of the scaled up batch was found to be 158.5 ± 9.8 nm and the drug loading was determined to be 10.32 ± 1.4%. The *in vitro* release study illustrated a slow sustained release corresponding to 75% drug over a period of 10 days. The pharmacokinetic profile of PLGA-CURC in rats following i.v. administration showed two compartmental model with the area under the curve (AUC_0-∞_) being 6.139 mg/L h. Gamma sterilization showed no significant change in the particle size or drug loading of the nanoparticles. Stability analysis revealed long term physiochemical stability of the PLGA-CURC formulation.

**Conclusions:**

A successful effort towards formulating, optimizing and scaling up PLGA-CURC by using Solid-Oil/Water emulsion technique was demonstrated. The process used CCD-RSM for optimization and further scaled up to produce 5 g of PLGA-CURC with almost similar physicochemical characteristics as that of the primary formulated batch.

## Introduction

Curcumin is one of the most promising natural anti-cancer agents and hence been much investigated for the past few decades [1,2]. Several phase I and phase II clinical trials indicate that curcumin is quite safe and may exhibit therapeutic efficacy [3-5]. A purified form of curcumin which consists of three main components: curcumin (76.07%); bisdemethoxy curcumin (3.63%); and demethoxy curcumin (20.28%) is defined as curcumin C3 complex [6]. Henceforth, curcumin C3 complex will be referred to as curcumin in this paper. Poor water solubility, poor physiochemical properties and low bioavailability continue to pose major challenges in developing a curcumin formulation for clinical efficacy. Lower serum and tissue levels of curcumin are observed irrespective of the route of administration due to extensive intestinal and hepatic metabolism and rapid elimination, thus restraining bioavailability of curcumin [7-10]. To improve its potential application in the clinical arena, several formulation strategies like nanoparticles, liposomes, complex with phospholipids, cyclodextrins and solid dispersions are being developed to improve bioavailability of curcumin and increasing its therapeutic efficacy [10-17]. Among these approaches, biodegradable polymeric nanoparticle based delivery systems offer significant advantage over other nanocarrier platforms as there is tremendous versatility in the choice of polymer matrices that can be used for tailoring nanoparticle properties to meet various drug delivery needs.

Although much research emphasis are presently being dedicated to various nanoparticle formulations in the pharmaceutical industry, especially towards particle design and targeting, very few results have ever been published on process scale-up. Scaling up of the formulation process is essential for clinical use. In this paper, we have made an effort towards optimizing and scaling up PLGA nanoparticles encapsulating curcumin (PLGA-CURC) by using Solid-Oil/Water (S-O/W) an emulsification-solvent-evaporation/diffusion technique. The major goals in designing polymeric nanoparticles as a delivery system are to control particle size and polydispersity, maximize drug encapsulation efficiency and drug loading, optimize surface properties and tailor release of pharmacologically active agents to achieve a site specific action of the drug at the therapeutically optimal desired rate and dose regimen [18,19]. Optimization becomes especially important when the formulation needs to be scaled up for industrial production. The organic solvent used in the formulation becomes critical for pilot and industrial scale production and hence only class 3 solvents are preferred for formulation while scaling up. In our formulation, we used ethyl acetate as the organic solvent. Partially hydrolyzed PVA was used as emulsion stabilizer as it prevents redispersibility problems [20].

For the optimization process, our aim was to use Response Surface Methodology (RSM) in conjunction with Central Composite Design (CCD) to establish the functional relationships between three chosen operating variables: polymer (PLGA) concentration, stabilizer (PVA) concentration and volume of organic phase (ethyl acetate). Four responses were identified namely, mean particle size, polydispersity, encapsulation efficiency (EE) and drug loading (DL) of PLGA-CURC for this study. The optimization procedure involved systematic formulation designs to minimize the number of trials, and analyze the response surfaces in order to realize the effects of factors and to obtain the appropriate formulations with target goals [21,22]. Further, for analyzing the responses to the variables, mathematical model equations were derived by using Design-Expert® 5.0 software. For a better understanding of the three variables or the optimal PLGA-CURC performance, the models were presented as three-dimensional contour response surface graphs.

Once the optimized batch was determined, classical scale up was followed to produce gram amounts of nanoparticle formulation. The nanoparticles obtained from the scale up were then characterized for particle size, polydispersity, drug loading and morphology and compared with non-scaled up optimized batch, thereby establishing successful process scale-up. Nanoparticles from the scaled up batch were further evaluated for percentage cumulative release, functional assays, cellular uptake in different cancer cell lines and storage stability.

## Materials and methods

### Materials

Poly (D,L-lactide-co-glycolide) 50:50; i.v. 0.77 dL/g (~0.5% w/v in chloroform at 30^o^ C); m.w. 124 kDa was purchased from Lakeshore Biomaterials (Birmingham, AL). Curcumin c3 complex was a kind gift from Sabinsa Corporation (East Windsor,NJ), Polyvinyl alcohol (m.w. 9,000-10,000; 80% hydrolyzed), ethyl acetate, ethanol, nile red, D(+) trehalose, sucrose, were purchased from Sigma Aldrich (St. Louis, MO). The human prostate cancer cell line - DU 145, breast cancer cell line - MDA-MB-231 and pancreatic cancer cell line MiaPaCa were obtained from ATCC (Manassas, VA). RPMI 1640, DMEM and FBS was obtained from Gibco, Invitrogen (Carlsbad, CA). Gold antifade mounting agent with 4’-6-diamidino-2-phenylindole (DAPI) was purchased from Invitrogen (Carlsbad, CA). Double-distilled deionized water was used for all the experiments.

### Methods

#### PLGA-CURC preparation

**Figure 1:**
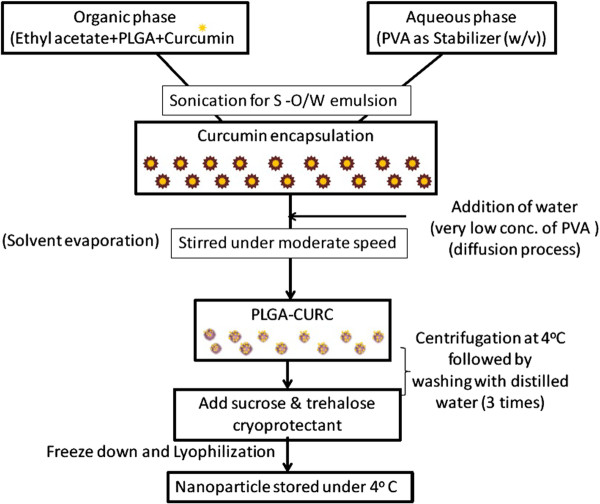
Schematic representation of the experimental procedure for the formulation of PLGA-CURC.

PLGA-CURC was prepared by S-O/W, an emulsification-solvent evaporation/diffusion method. The method is ideal for encapsulation of hydrophobic compounds like curcumin. Briefly, the polymer PLGA (mg) was dissolved in ethyl acetate. Curcumin (15% w/w) was added and allowed to dissolve with intermittent vortexing using Vortex Mixer (Fisher Scientific, Vortex Genie 2 G-560, Scientific industries Inc, Bohemia, NY). Solid-in-oil mixture was added to an aqueous phase of PVA (w/v) to form S-O/W emulsion. Once all the drug/polymer mixture was added to the PVA solution, the contents were vortexed for 10 sec at a high setting. The resulting suspension was sonicated for 60 sec at 45% amplitude with a sonic disrupter (Hielscher UP200S ultrasonic processor, Ringwood, NJ). Immediately after sonication, the emulsion was poured into to excess of aqueous phase (0.1% PVA in water; 40 ml) for diffusion under rapid stirring with a magnetic stirrer. This colloidal suspension was kept on a magnetic stirrer for complete solvent evaporation for 5–6 h. The nanoparticles were then collected by centrifugation, washed 3 times with distilled Milli Q treated water. Finally, they were resuspended in 2 ml of cryoprotectent solution (sucrose (2 %w/w) and trehalose (5% w/w)), dried on a lyophilizer ATR FD 3.0 system (ATR Inc., St Louis, MO) and stored at 4°C. Figure 1 shows the schematic representation of the experimental procedure for the PLGA-CURC formulation.

#### Experimental design for optimization of formulation

The most efficient way to test different variables simultaneously requires a systematic and detailed experimental design. This eliminates the need for a large number of independent runs when the classical step-by-step method is used. Optimization procedures like RSM run by selecting an objective function, finding the contributing factors and investigating the relationship between responses and factors [23]. Preliminary experiments indicated that variables such as amount of polymer PLGA, PVA concentration and volume of ethyl acetate were the main factors that affected the particle size, size distribution, percentage drug loading and encapsulation efficiency of the PLGA-CURC. A CCD model was used to statistically optimize the formulation parameters and evaluate the main effects, interaction effects and quadratic effects of the formulation factors on the particle size (*Y*_1_), size distribution (*Y*_2_), percentage drug loading (*Y*_3_) and encapsulation efficiency (*Y*_4_) of PLGA-CURC. Details of the design are listed in Table 1. For each factor, the experimental range was selected on the basis of the results of preliminary experiments and the feasibility of preparing the PLGA-CURC at the extreme values. The value range of the variables was: amount of PLGA (X_1_): 50–120 mg, PVA concentration (X_2_): 1.0–3.0%, and volume of ethyl acetate (X_3_): 2.0–5.0 ml. The design consists of 15 runs (8 factorial points, 6 star points and 1 center point) and 5 replicated runs (center points) yielding 20 experiments in total (Table 2). The purpose of replication was to estimate experimental error and increase the precision. Each experimental run was repeated thrice. The star points represents extreme values (low and high) for each factor in the design and allow for estimation of second-order effects. The star points are at some distance, alpha, from the center based on the properties desired for the design and the number of factors in the design. Alpha in coded units is the axial distance from the center points and makes the design rotatable. A rotatable design provides equally good predictions at points equally distant from the center, a very desirable property for Response Surface Methodology. 

**Table 1:** Relationship between coded and actual values of the variables used for PLGA-CURC formulation

**Formulation factors**	**Coded level of variables**				
	**-α**	**−1**	**0**	**1**	**+α**
*X*_1_ = Amount of PLGA (mg)	26.14	50	85	120	143.86
*X*_2_ = PVA (% w/v)	0.32	1.0	2.0	3.0	3.68
*X*_3_ = Ethyl acetate (ml)	0.98	2	3.5	5.0	6.02
**Dependent variables**		**Constraints**			
*Y*_1_ = Particle Size (nm)		Minimize			
*Y*_2_ = Polydispersity		Minimize			
*Y*_3_ = Encapsulation efficiency (%)		Maximize			
*Y*_4_ = Drug loading (%)		Maximize			

**Table 2:** Observed responses in central composite design for PLGA-CURC formulation

**Exp. No**	**Wt. of PLGA (mg)**	**PVA conc. (% w/v)**	**Ethyl Acetate (ml)**	**Particle Size (nm)**	**Polydispersity**	**Encapsulation efficiency (%)**	**Drug loading (%)**
1	50	1	2	125.6	0.135	73.4	11.1
2	120	1	2	142.5	0.148	63.35	11.4
3	50	3	2	108.5	0.125	73.84	11.3
4	120	3	2	144.5	0.143	63.86	13.5
5	50	1	5	81.5	0.097	83.25	14.9
6	120	1	5	133.8	0.116	85.5	13.2
7	50	3	5	103.7	0.115	83.54	10.5
8	120	3	5	136.7	0.135	84.8	15.8
9	26.14	2	3.5	133.3	0.138	63.25	5.5
10	143.86	2	3.5	198.8	0.172	53.46	9.4
11	85	0.32	3.5	158.8	0.164	88.85	13.6
12	85	3.68	3.5	149.5	0.155	88.25	13.8
13	85	2	0.98	176.9	0.093	53.35	14.5
14	85	2	6.02	77.8	0.067	63.78	15.2
15	85	2	3.5	112.5	0.135	86.95	11.8
16	85	2	3.5	121.6	0.148	90.35	12.6
17	85	2	3.5	119.8	0.135	91.46	10.5
18	85	2	3.5	118.6	0.147	93.54	11.9
19	85	2	3.5	129.6	0.128	90.34	12.8
20	85	2	3.5	119.7	0.172	86.27	12.9

A design matrix comprising of 20 experimental runs was constructed and the responses were modeled by the following polynomial model:

(1) $$y=b_{0}+b_{1}x_{1}+b_{2}x_{2}+b_{3}x_{3}+b_{11}x_{1}^{2}+b_{22}x_{2}^{2}+b_{33}x_{3}^{2}+b_{12}x_{1}x_{2}+b_{13}x_{1}x_{3}+b$$

Where Y is the measured response associated with each factor level combinations; *b*_o_ is the Intercept; *b*_i_ ‘s (for *i* = 1,2, and 3) are the linear effects, the *b*_ii_ are the quadratic effects, the *b*_ij_ ‘s (for *i,j* = 1,2,and 3, *i* < *j*) are the interaction between the *i*_th_ and *j*_th_ variables.

Data were analyzed by using analysis of variance (ANOVA), which helped determine if the factors and the interactions between factors were significant. To test whether the terms were statistically significant in the regression model, t-tests were performed using a 95% (α = 0.05) level of significance. An F-test was used to determine whether there was an overall regression relationship between the response variable Y and the entire set of X variables at a 95% (α=0.05) level of significance. The coefficient of multiple determinations was denoted by R^2^, which measured the proportionate reduction of total variation in Y associated with the use of the set of X variables. In addition, the validity of the regression model was assessed according to statistical assumptions and lack of fit test. The statistical analysis was performed using the software Design Expert (Version 5) (Stat-Ease, Inc, Minneapolis, MN).

#### Determination of desirability coefficient

All four responses studied are critical for the optimization of PLGA-CURC formulation. However, it is difficult to optimize all the objectives simultaneously because they do not coincide with each other and conflict may occur between them. The optimum condition reached in one response may have an opposite influence on another. For finding the best optimized formulation for all responses, the multi criteria problem can be treated as single criterion by using the desirability function approach. This was performed in two steps. A predictive model for the response of performance parameters in the formulation of nanoparticle was first obtained by an analysis of variance and then a desirability index for each response was evaluated using the software Design Expert. The individual desirability indices were then used to construct the combined objective function called the desirability coefficient, which is the geometric mean of all the transformed responses and is given by the Eq. 2 [24].

(2) $$\delta ={\left(d_{1}\times d_{1}\times \cdots \cdots d_{n\;}\right)}^{1/n}$$

where *d*_i_’s are values obtained by transforming the measured response based on the desired goal. Hence, when the goal was to maximize a certain response, the *d*_i_ values were defined as

(3) $$d_{i}={\left\{\frac{y_{i}-y_{i}^{\min}}{y_{i}^{\max}-y_{i}^{\min}}\right\}}^{w_{i}}$$

where w_i_ is the weight index and *y*_i_^max^ and *y*_i_^min^ are the maximum and minimum values of the responses used for calculating *d*. When the goal was to minimize a response, the *d*_i_ values were defined as

(4) $$d_{i}={\left\{\frac{y_{i}^{\max}-y_{i}}{y_{i}^{\max}-y_{i}^{\min}}\right\}}^{w_{i}}$$

One may look upon the *d*_i_ as the value of the response on a new scale between zero and unity. The exponent (weighting factor) defines curvature of the interpolation equation. For example, when *w*_i_ = 1, the interpolation is linear. Since the *d*_i_ values are in the range 0 ≤ *d*_i_ ≤1, the desirability coefficient is also in the range 0 ≤ *δ* ≤1. The index *n* equals ∑*w*_i_. The contour plot of desirability coefficients reported here is based on the *d*_i_ values computed for four variables, namely, for particle size (*d*_*1*_), polydispersity (*d*_*2*_), encapsulation efficiency (*d*_*3*_) and drug loading (*d*_*4*_). The goals considered were minimizing particle size and polydispersity, maximizing encapsulation efficiency and drug loading. The desirability coefficient *δ* was computed in this fashion and the contours of equal *δ* values were plotted. To obtain the condition on the design variables that maximize *δ*, a three-dimensional graph of the response against any two factors was plotted, from which the region corresponding to optimum values for *δ* was yielded.

### Characterization of PLGA-CURC

#### Particle size and polydispersity

Particle size measurements and polydispersity of PLGA-CURC were determined by laser diffraction using a Nanotrac system (Mircotrac, Inc., Montgomeryville, PA). Lyophilized PLGA-CURC were dispersed in double distilled water as described elsewhere [14,25] and analyzed in triplicates with three readings per nanoparticle sample. The polydispersity was also calculated based on the volumetric distribution of particles.

### Determination of curcumin associated with PLGA-CURC

Lyophilized PLGA-CURC (5 mg) was dissolved in 2 ml acetonitrile to extract curcumin into acetonitrile for determining the encapsulation efficiency. The samples in acetonitrile were gently shaken on a shaker for 4 h at room temperature to completely extract out curcumin from the nanoparticles into acetonitrile. These solutions were centrifuged at 14,000 rpm (Centrifuge 5415D, Eppendorf AG, Hamburg, Germany) and supernatant was collected. Suspension (20 μl) was dissolved in ethanol (1 ml) and was used for the estimations. The curcumin concentrations were measured spectrophotometrically at 450 nm. A standard plot of curcumin (0–10 μg/ml) was prepared under identical conditions.

The encapsulation efficiency (EE) of PLGA-CURC was calculated using

(5) $$Encapsulation\;efficiency\;\left(\%\right)=\frac{Total\;drug\;content\;in\;nanoparticles}{Total\;drug\;amount}\times 100$$

#### Percentage drug loading

The percent drug loading was calculated by total amount of drug extracted from the polymeric nanoparticles to the known weight of nanoparticles

(6) $$Drug\;loading\;\left(\%\right)=\frac{Drug\;content}{Weight\;of\;nanoparticles}\times 100$$

#### *In vitro* drug release study

The *in vitro* drug release profiles of optimized PLGA-CURC formulations were determined by measuring the cumulative amount of drug released from the nanoparticle over predetermined time intervals as described elsewhere [14].

To study and better understand the release mechanism of curcumin from nanoparticle formulation, data obtained from *in vitro* drug release studies were fitted in different kinetic models [26,27]. For zero order, cumulative amount of drug released was plotted versus time; for first order, log cumulative percentage of drug remaining was plotted versus time; for Higuchi’s model, cumulative percentage of drug released was plotted versus the square root of time and for Hixson–Crowell cube root model, cumulative percentage of drug release was plotted versus cube root of time. Plotted data were fitted using a linear equation; the regression coefficient (r^2^) was calculated from the appropriate graphs. Selection of the best model was based on the comparisons of the relevant correlation coefficients.

### External morphology studies

#### Transmission electron microscopy (TEM)

The surface morphology of the nanoparticles was studied using transmission electron microscopy (TEM) as described elsewhere [14,25].

#### Scanning electron microscopy (SEM)

The surface morphology of the formulated nanoparticle was measured by scanning electron microscopy (SEM) (EM- LEO 435VP, Carl Zeiss SMT Inc., NY) equipped with 15 kV, SE detector with a collector bias of 300 V. The lyophilized samples were carefully mounted on an aluminum stub using a double stick carbon tape. Samples were then introduced into an automated sputter coater and coated with a very thin film of gold before scanning the samples under SEM.

#### *In vitro* cellular uptake of PLGA-CURC in cancer cells

Curcumin is intrinsically fluorescent; this facilitated the visualization of the PLGA-CURC uptake into cells under confocal microscope. To observe the internalization of nanoparticles under a confocal microscope, DU-145, MDA-MB 231 and MiaPaCa were grown under standard cell culture conditions. Cellular uptake of Nile red-labeled PLGA-CURC was determined using a confocal microscope (Zeiss LSM 510 META attached to a Zeiss Axiovert 200 inverted microscope) (Carl Zeiss MicroImaging, Inc., Thornwood, NY). For these experiments, cells were placed on a cover slip in a 6-well tissue culture plate and incubated at 37°C until they reached sub-confluent levels. The cells were then exposed to 1 mg/ml concentrations of nile red labled PLGA-CURC. After 2 h, the treated cells were fixed with standard paraformaldehyde (4%) and fixed using Gold antifade mounting agent with 4’-6-diamidino-2-phenylindole (DAPI). The slides were viewed under the microscope to determine the extent of intracellular nanoparticle uptake.

#### Western blot analysis to determine the functional integrity

For Western blot, 30–50 μg of nuclear and cytoplasmic and protein extracts, prepared by the nuclear extraction kit (Pierce, USA) protocol, were resolved on 10% SDS-PAGE gel. After electrophoresis, the proteins were electrotransferred to a nitrocellulose membrane, blocked with 5% non-fat milk, and probed with antibodies against the p65 subunit of NFκβ. Thereafter, the blot was washed, exposed to HRP-conjugated secondary antibodies for 1 hour, and finally detected by ECL chemiluminescence reagents (Amersham Pharmacia Biotech, Arlington Heights, IL).

#### Scale up for large batch of nanoparticles production

The process scale up of PLGA-CURC formulation was a multistage process wherein each stage was optimized. In the first stage, we expected to scale up 5X to produce 500 mg of PLGA-CURC. In the second stage, 1 g production was targeted. Subsequent stages would lead to 2 g and 5 g (50X) production of PLGA-CURC. For scaling up, we reduced the ratio of organic phase and aqueous phase else the amount of organic solvent would be very high and accordingly adjusted other parameters to keep similar nanoparticle characteristics. All the parameters which were varied at each stage of scale up are listed in Tables 3 and 4.

**Table 3:** Formulation factors and parameter variations for process scale up

	**PLGA (mg)**	**PVA %(w/v)**	**Ethyl acetate (ml)**	**Aqueous phase (ml)**	**Excess Water (ml)**	Sonication Tip*	**Sonication Time (s)**	**Stirrer Speed (rpm)**	**Stirrer Time (h)**
**Primary optimized conditions**	85	1	4.25	15	40	S2	60	2000	4
**First stage – 500 mg**	500	1	5	20	50	S2	120	2000	4
**Second stage – 1 g**	1000	1	10	40	60	S14	120	3000	6
**Third stage- 2 g**	2000	1	20	60	80	S14	180	6000	6
**Fourth stage – 5 g**	5000	1	50	150	200	S14	300	8000	6

*S2 – soncation tip dia – 2 mm, S14 – sonication tip dia – 14 mm.

**Table 4:** Scale up results for large batch production of PLGA-CURC

	**Particle size (nm)**	**Polydispersity index**	**Encapsulation efficiency (%)**	**Drug loading (%)**
**Primary optimized conditions**	129.5 ± 6.9	0.138 ± 0.023	91.4 ± 2.3	12.68 ± 3.5
**First stage – 500 mg**	135.4 ± 9.4	0.139 ± 0.012	91.12 ± 1.5	11.98 ± 2.5
**Second stage – 1 g**	142.3 ± 8.9	0.142 ± 0.024	92.13 ± 3.5	11.12 ± 2.6
**Third stage- 2 g**	148.6 ± 7.7	0.137 ± 0.025	90.67 ± 2.8	10.92 ± 2.3
**Fourth stage – 5 g**	158.5 ± 9.8	0.141 ± 0.011	90.34 ± 3.2	10.32 ± 1.4

#### Pharmacokinetic studies

Pharmacokinetic studies were carried out to analyze the bioavailability of curcumin following intravenous (i.v.) administration of PLGA-CURC. For this study, male Sprague Dawley rats weighing 250–300 g were used in a protocol approved by the IACUC committee of UNTHSC, Fort Worth, Texas, USA. Eight animals were administered PLGA-CURC (7.5 mg curcumin equivalent/kg of bodyweight) by tail vein injection. Blood samples (200μL) were collected into heparinized microcentrifuge tubes at predetermined time points. After each blood sampling, same amount of normal saline was administered to compensate for the blood loss. Plasma was separated by centrifuging the blood samples at 3,500 rcf for 10 min at 4°C. To 100 μL aliquot of acetonitrile containing 0.15 μg/mL of internal standard was added to 100 μL of each plasma samples in order to precipitate the plasma protein. Samples were vortexed for 5 min and then subjected to centrifugation at 2,500 rcf for 15 min to remove any precipitated material. Finally, samples were injected into the HPLC system through the autosampler. The concentration of curcumin was determined by HPLC analysis and quantitated with previous calibrations [28].

#### Stability studies

PLGA-CURC (20 mg) was kept in four sealed glass vials and maintained at 4°C for a period of 6 months. Nanoparticles were characterized for change in particle size, encapsulation efficiency and percent drug loading according to the above mentioned protocols.

#### Gamma irradiation of nanoparticles

Gamma irradiation is recommended by European Pharmacopoeia for the purpose of sterilizing pharmaceutical products. Such studies for our nanoparticles were carried out by Steris Isomedix Services, IL, USA. Drug loaded nanoparticles were γ-irradiated using ^60^Co as irradiation source and received a dose of either 16.8 kGy for 241 minutes (Low), 25.3 kGy for 179 minutes (Medium) or 35.8 kGy for 241 minutes (High). Non-irradiated samples were kept as reference for further comparison.

## Results and discussion

### Optimization of PLGA-CURC using central composite design

Response Surface Methodology (RSM) using the Central Composite Design (CCD) model is a well-suited experimental design strategy that offers the possibility of investigating a high number of variables at different levels with only a limited number of experiments [29]. The methodology was originally developed by Box and Wilson and improved by Box and Hunter. This is an ideal tool for process optimization [23], and its rotatable characteristic enables identification of optimum responses around its center point without changing the predicting variance. RSM is a collection of mathematical and statistical techniques based on the fit of a polynomial equation to the experimental data, which must describe the behavior of a data set with the objective of making statistical provisions. CCD has been successfully used to optimize the technology or production condition for drug delivery systems such as sustained release tablets, liposomes, microspheres, nanoparticles in recent years [29-35]

The ranges for each of the variables in Table 1 were chosen taking into account our preliminary experiments. Table 2 shows the experimental results concerning the tested variables on mean diameter of particle size, polydispersity drug loading percentage and encapsulation efficiency. These four responses were individually fitted to a second order polynomial model. For each response, the model which generated a higher F value was identified as the best fitted model. Each obtained model was validated by ANOVA. Three dimensional response surface plots were drawn for the optimization of PLGA-CURC formulation. These types of plots are useful in studying the effects of two factors on the response at one time, when the third factor is kept constant.

### Influence of formulation variables on particle size

Particle size is a critical factor for nanoparticle-based drug delivery system. It is one of the factors that control the kinetics of drug release. Generally, smaller particle size permits a faster release rate. The following second order reduced quadratic model equation was derived by the best fit method to describe the relationship between the particle size (Y_1_), the amount of PLGA, concentration of PVA and volume of ethyl acetate.

(7) $$\begin{array}{l} Particle\;size(Y_{1})=180.43-1.35^{*}PLGA-59.48^{*}PVA+45.26^{*}Ethyl\;acetate+11.85^{*}\\ PVA^{2}-9.16^{*}Ethyl\;acetate^{2}+0.077^{*}PLGA^{*}Ethyl\;acetate+3.35^{*}PVA^{*}Ethyl\;acetate \end{array}$$

A positive value in regression equation for a response represents an effect that favors the optimization (synergestic effect), while a negative value indicates an inverse relationship (antagonistic effect) between the factors and the response [30].

The reduced quadratic model was found to be significant with an *F* value of 30.87 (p < 0.0001), which indicates that response variable Y_1_ and the set of formulation variables were significantly related. The high R^2^ value indicated that 96.53% of variation in particle size was explained by the regression on formulation factors (Additional file 1: Table S1).

**Figure 2:**
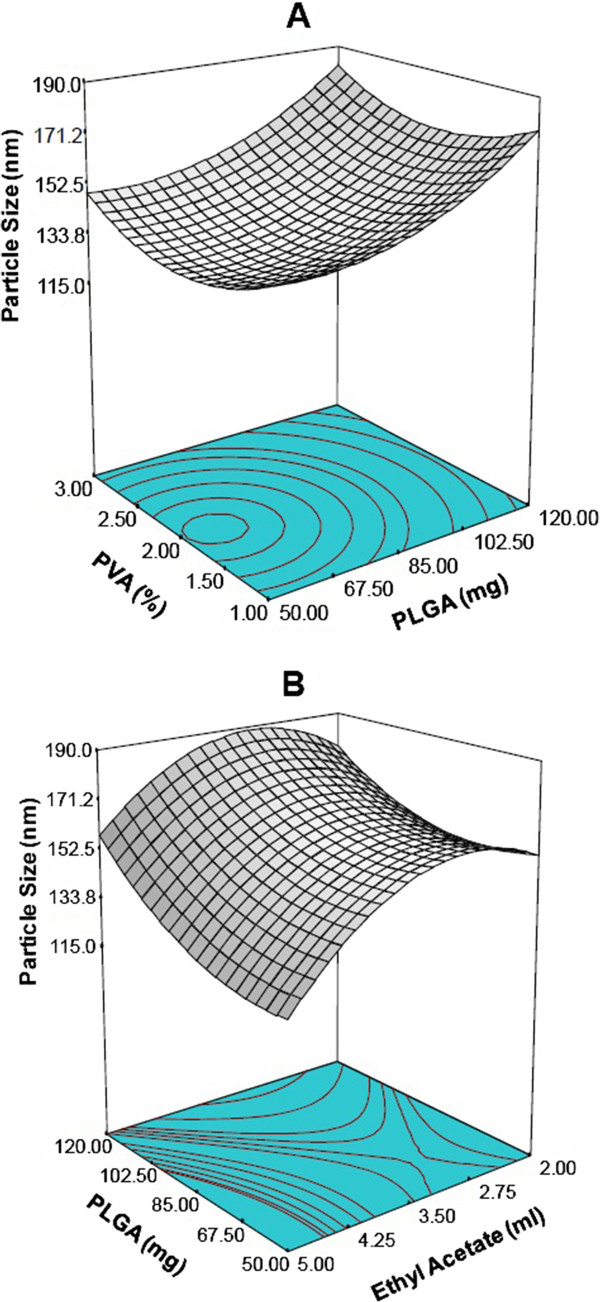
Three dimensional response surface plots showing the effect of variables on response:- particle size; (A) effect of PLGA and PVA concentration on particle size (Actual constant ethyl acetate (ml) = 4.25); (B) effect of PLGA and ethyl acetate on particle size (Actual constant PVA (%) = 1.0).

The particle size values for the 20 batches show a wide variation in response i.e., the response ranges from a minimum of 77.8 nm to a maximum of 198.8 nm. The data clearly indicate that the particle size value is strongly dependent on the selected variables. The response surface plots for particle size as a function of formulation factors were constructed by holding one of the factors at a constant level. Figure 2A shows the response surface plot obtained for the interaction between PLGA concentration and PVA at constant value of ethyl acetate. An increase of the mean particle size was observed (Figure 2A) when increasing concentration of PLGA for all the amount of PVA used in the formulation (1–3%). It was reported that an increase in the amount of PVA in the formulation may lead to the smaller particle size due to tight surface that was formed from PVA macromolecular chains of high concentration [36]. However, too much PVA is not suggested as it will hinder *in vivo* degradation*.* In addition, PVA has been found to have a carcinogentic potential and removal of excess PVA from the particle surface is difficult [37]. Our data support that a lower concentration of PVA (1% w/v) was suitable to obtain well controlled particle size formulations. Analyzing the response surface of interactions of PLGA and ethyl acetate at constant PVA, we found that initially, with an increase of solvent volume, the particle size does not change much and then decreases with further increase in the volume of ethyl acetate (Figure 2B). Formation of nanoparticles depends on the rate of diffusion of the organic solvent into the aqueous phase, which in turn influences the precipitation of polymer thereby influencing the particle size. The minimum particle size and its corresponding experimental conditions were derived from the regression model. 

### Influence of preparation factors on polydispersity index

**Figure 3:**
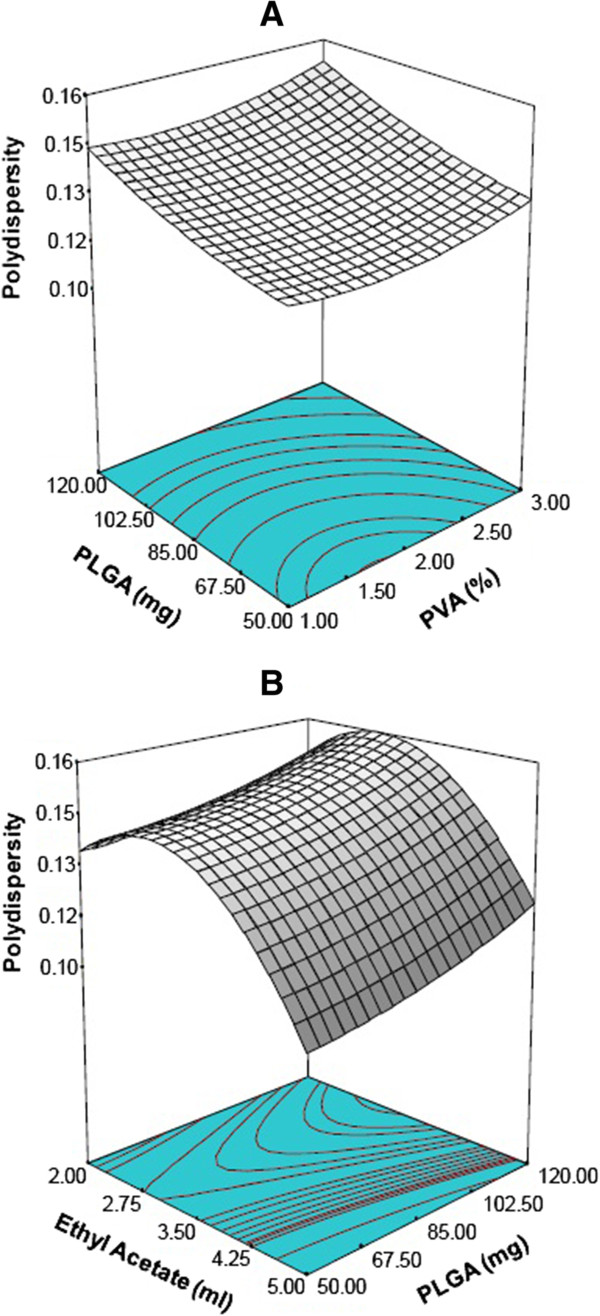
Three dimensional response surface plots showing the effect of variable on response:- polydispersity; (A) effect of PLGA and PVA concentration on polydispersity (Actual constant ethyl acetate (ml) = 4.25); (B) effect of PLGA and ethyl acetate on polydispersity (Actual constant PVA (%) = 1.0).

After nanoparticle formation, the size population frequently follows a multimodal distribution. The polydispersity index is a very important parameter which is used to describe variation of particle size in a sample of particles. When this index is close to 1, the size range becomes wide. A desired optimal value is closer to 0. The response surfaces of polydispersity index keeping ethyl acetate and PVA constant are shown in Figure 3A and B respectively. The polydispersity variations are found to be in the same direction as the particle size in all the cases studied. Increasing the amount of polymer and decreasing the volume of organic phase leads to an increase of the polydispersity index. The coefficient of correlation, R^2^ is 0.8927 and the model gives a p-value <0.001 (ANOVA).

(8) $$\begin{array}{l} Polydispersity=0.091-0.00026^{*}PLGA-0.035^{*}PVA+0.056^{*}Ethyl\;acetate+0.00458^{*}\\ PVA^{2}-0.010^{*}Ethyl\;acetat{e^{2}}^{*}Ethyl\;acetate+0.00433^{*}PVA^{*}Ethyl\;acetate \end{array}$$

The minimum polydispersity index and its corresponding experimental conditions were derived from this regression model. The values of polydispersity index predicted from this regression model are shown in Table 5.

**Table 5:** Comparison of the experimental and predicted values of PLGA-CURC prepared under the predicted optimum conditions

**Response**	**Predicted value**	**Experimental value**	**Bias (%)**^**a**^
*Y*_*1*_, Particle size (nm)	120.73	129.5	6.77
*Y*_*2*_, Polydispersity index	0.13	0.138	6.15
*Y*_*3*_, Encapsulation efficiency (%)	92.45	91.4	−1.13
*Y*_*4*_, Drug loading (%)	13.56	12.68	−6.49

^a^ Bias was calculated as (predicted value-experimental value)/predicted value X 100.

### Influence of preparation factors on encapsulation efficiency

**Figure 4:**
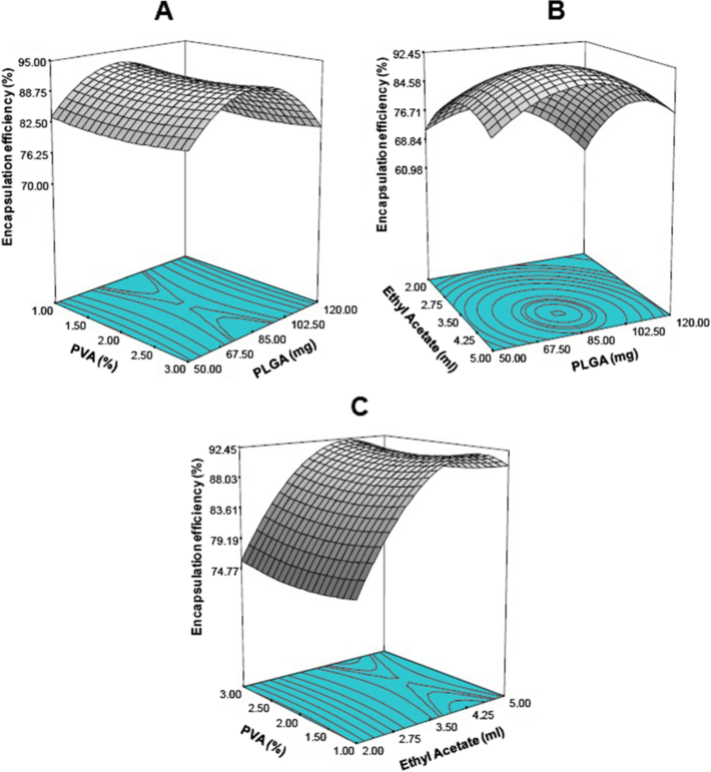
Three dimensional response surface plots showing the effect of variable on the response:- encapsulation efficiency; (A) effect of PLGA and PVA concentration on encapsulation efficiency (Actual constant ethyl acetate (ml) = 4.25); (B) effect of PLGA and ethyl acetate on encapsulation efficiency (Actual constant PVA (%) = 1.0); (C) effect of PVA and ethyl acetate on encapsulation efficiency (Actual constant PLGA (mg) = 4.5).

In our study, encapsulation efficiency of PLGA-CURC reached up to 93.5% (Table 2). High encapsulation efficiency is advantageous since it transports enough drug at the target site and increase the residence time of the drug. The high encapsulation efficiency in PLGA can be attributed to several factors. First the hydrophobic nature of PLGA molecules makes it relatively easy to entrap hydrophobic curcumin into PLGA-CURC. Second, the hydrophobic nature of curcumin results in a minimum loss of the drug to the external aqueous phase during the formulation process. The response surface diagrams reveal that the encapsulation efficiency first increases with increasing PLGA concentration and then decreases (Figure 4A and B) at constant PVA and ethyl acetate concentration. Furthermore, there is no significant change observed with variation of PVA concentration (1–3% w/v) (Figure 4C). The optimized variables show a good fit to the quadratic model (Eq. 9) with an *F* value of 14.15 (p =0.0001), which indicates that response variable Y_3_ and the set of formulation variables were significantly related. The high R^2^ value indicated that 92.73% of variation in encapsulation efficiency was explained by the regression on formulation factors (Additional file 1: Table S1).

The statistical analysis of the results generated a quadratic response for encapsulation efficiency is as follows

(9) $$\begin{array}{l} Encapsulation\;efficiency\;\left(\%\right)=0.54+1.07^{*}PLGA-3.88^{*}PVA+24.17^{*}Ethyl\;acetate+\\ 1.13^{*}PVA^{2}-3.42^{*}Ethyl\;acetate^{2}+0.056^{*}PLGA^{*}Ethyl\;acetate-0.11^{*}PVA^{*}Ethyl\;acetate \end{array}$$

### Influence of preparation factors on percentage drug loading

**Figure 5:**
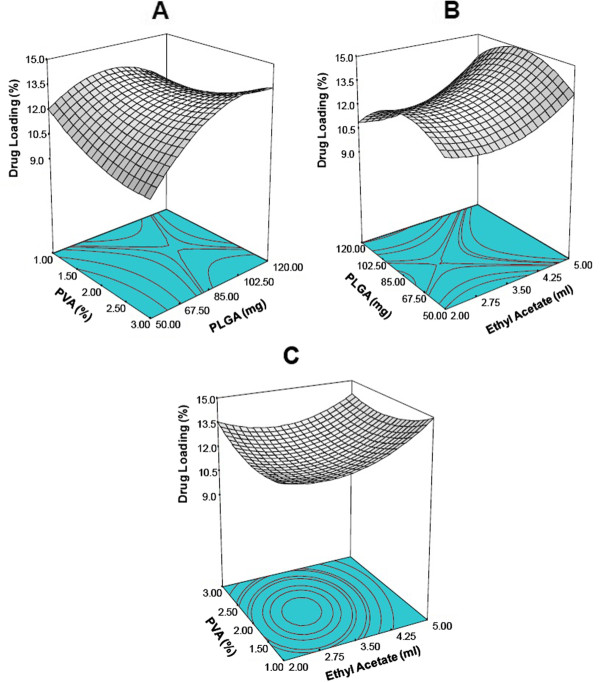
Three dimensional response surface plots showing the effect of variable on response:- drug loading; (A) effect of PLGA and PVA concentration on the drug loading (Actual constant ethyl acetate (ml) = 4.25); (B) effect of PLGA and ethyl acetate on drug loading (Actual constant PVA (%) = 1.0); (C) effect of PVA and ethyl acetate on drug loading (Actual constant PLGA (mg) = 4.5).

The response surface graphs for the most statistically significant variables on percentage drug loading are shown in Figure 5(A-C). The response surface diagram depicting interactions of PLGA concentration and PVA showed that increase in polymer concentration first increases the percentage drug loading and then decreases implying an optimum polymer concentration for maximum drug loading. At higher PLGA concentration, initially there was no significant change observed for drug load with respect to PVA concentration, but drug loading increased with increase in PVA concentration. The reverse was observed at low PLGA concentration.

(10) $$\begin{array}{l} Drug\;loading\;\left(\%\right)=14.34+0.16^{*}PLGA-4.34^{*}PVA-2.65^{*}Ethyl\;acetate+0.72^{*}PVA^{2}+\\ 0.50^{*}Ethyl\;acetate^{2}+0.032^{*}PLGA^{*}PVA-0.34^{*}PVA^{*}Ethyl\;acetate \end{array}$$

### Optimization by desirability function

**Figure 6:**
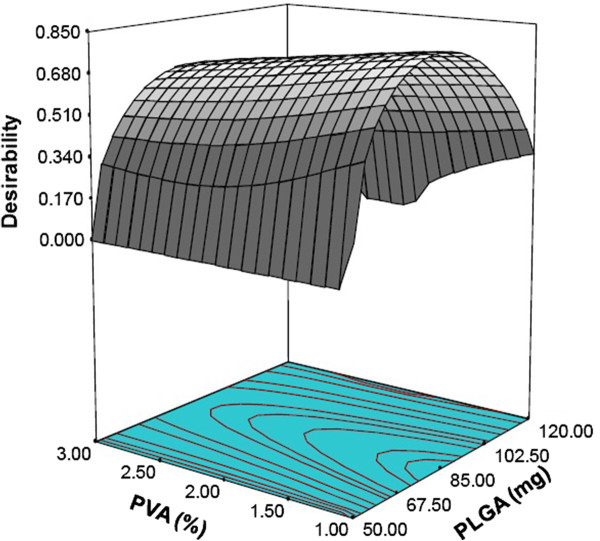
Response surface for overall desirability (δ as a function of PLGA (mg) and PVA (%) at constant ethyl acetate at 4.25 ml.

Optimization process was undertaken with desirability function to optimize the four responses simultaneously. A high value of desirability coefficient *δ* (0 ≤ *δ* ≤ 1) indicates that the operating point can produce acceptable formulation results. The responses: particle size (Y_1_), polydispersity index (*Y*_2_), encapsulation efficiency (*Y*_3_) and drug loading (*Y*_4_) were transformed into the desirability scale *d*_1_, *d*_2_, *d*_3_ and *d*_4_, respectively. Among them, *Y*_1_, *Y*_2_ had to be minimized, while *Y*_3_, *Y*_4_ had to be maximized. The overall objective function (*δ*) was calculated by Eq. (2). The model was fitted with a second-order quadratic expression. The higher coefficient of determination and F value in terms of the quadratic model indicated the goodness of fit. Figure 6 shows the response surface plot for increasing desirability coefficient δ with respect to changes in variables: PLGA (*X*_1_) and PVA (*X*_2_) keeping the volume of ethyl acetate constant. The maximum value of desirability coefficient *δ* = 0.716 was obtained at the conditions, PLGA amount of 85 mg, PVA concentration of 1%(w/v) and 4.25 ml of ethyl acetate.

In order to evaluate the predictive power of this model and desirability coefficient, PLGA-CURC was prepared under the optimal conditions. The results comparing the experimentally obtained and model predicted values of all four responses are presented in Table 5. The experimental values of the multiple batches prepared under the optimal conditions were very close to the predicted values, with low percentage bias, suggesting that the optimized formulation was reliable and reasonable. It has been shown that the highest encapsulation efficiency and drug loading with commensurate minimum mean particle size and size distribution was achieved by using the optimal conditions of 85 mg PLGA, 1% (w/w) of PVA and 4.25 ml volume of ethyl acetate.

### Scale up for large batch production of PLGA-CURC

Scaling up the nanoformulation process to produce large batches of nanoparticles is the key to effective clinical use of nanoparticle based drugs. To translate this formulation into large scale production, we investigated seven critical parameters and their correlation in four sequential stages. Each stage was optimized to get the best parameter combination in terms to target response of particle size, polydispersity, encapsulation efficiency and drug loading. The parameters chosen include polymer solvent ratio, aqueous phase volume, organic and aqueous phase ratio, sonication tip diameter, sonication time, stirrer speed and stirring time. Our goal was to produce PLGA-CURC with similar physicochemical characteristics in a scaled up batch production. Results from all the different stages of scale up production are compiled in Tables 3 and 4 which shows the parameter variations and optimized scale up outcomes of PLGA-CURC formulation.

In the first scale-up optimization stage, polymer amount was increased from 85 mg to 500 mg but the volume of ethyl acetate was increased to only 5 mL from 4.25 mL. This was a very critical step as we needed to minimize the amount of organic solvent needed to prevent problems of solvent evaporation. This resulted in an increase in viscosity of the organic phase leading to larger particle size. To overcome this, the sonication time was increased from 60 sec to 120 sec keeping the sonication tip diameter and stirring speed and stirring time same. This resulted in average particle size of 135.4 nm, an increase of about 6 nm from the primary optimized batch. The drug loading in the first stage scale-up dropped by 0.7% while encapsulation efficiency remained almost the same. Once we achieved the first stage, next we scaled up ~10 times for producing 1 g of PLGA-CURC in the second stage. Doubling the amount of polymer and volume of solvents required increasing the sonication power to get nanoparticles in the same particle size range. For that, the sonication tip diameter was increased from 2 mm to 14 mm. Further, stirring speed was increased from 2,000 rpm to 3,000 rpm and stirring time was increased by 2 h. The resulting optimized batch had an average particle size of 142.3 nm and 11.12% of drug loading. In the third stage (~20X) of scale-up, the aqueous phase was optimized at 60 ml. To keep the nanoparticle size comparable, the sonication time was increased to 180 sec and the stirring speed was doubled to 6,000 rpm. Accounting for more than double of total volume from stage one to three, the exposed surface area for evaporation of solvents was increased by using an open mouthed vessel during stirring. With all these parameter combinations, the final optimized batch for this stage showed a 0.2% decrease in drug loading only and similar ~6 nm increase in particle size from previous stage. In the final stage, we scaled up to ~50 times to produce 5 g of PLGA-CURC. Here, the aqueous phase was increased to 150 mL with the excess water being increased to 200 mL to account for 5 g of polymer being used. Such a large volume of liquid phase needed high sonication power which was brought about by increasing the sonication time from 180 sec to 300 sec, increasing the stirring speed to 8,000 rpm and increasing the stirring time to 8 h. This resulted in nanoparticles having an average particle size of 158.5 nm, a total increase of only 29.4 nm from the primary optimized batch. Also, the drug loading decreased by 2.36% which is minimal considering ~50X scale-up. The encapsulation efficiency and polydipersity was found to be similar to the primary optimized batch. We have successfully produced PLGA-CURC in 5 g quantities through this route and identified critical parameters for scaling up the formulation process.

### Characterization and evaluation of the optimized scaled-up formulation

#### External morphology

**Figure 7:**
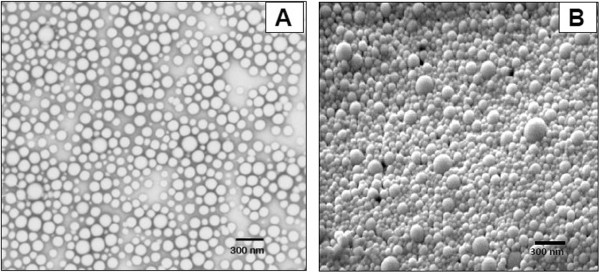
(A) Transmission electron micrograph and (B) Scanning electron micrograph of PLGA-CURC formulated under optimum conditions.

The external morphology of lyophilized PLGA-CURC prepared at optimal conditions are shown in Figure 7A and B. PLGA-CURC were spherical, discrete without aggregation, and smooth in surface morphology. The size of the PLGA-CURC was found to be approximately 140 nm. The particle size determined by Differential Light Scattering (DLS) for the same batch was found to be 158.5 nm. This may be explained by the fact that particle size analyzer, based on DLS, measures the hydrodynamic diameter of the particle while the electron microscope measures exact diameter of particles in solid state. But at the same time, the amount of nanoparticles seen under SEM or TEM is a very small random sample from the bulk of nanoparticle batch produced.

#### *In vitro* release studies for PLGA-CURC prepared under optimal conditions

**Figure 8:**
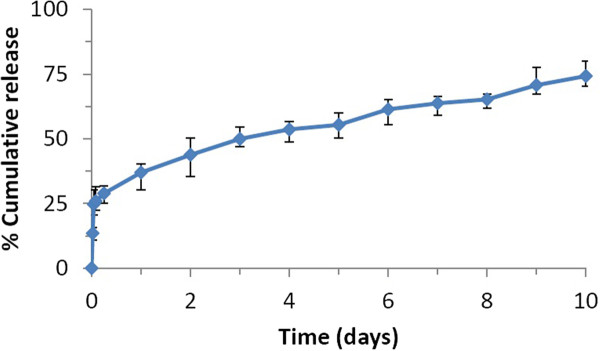
***In vitro*release profiles of the optimized formulations of PLGA-CURC (values reported are mean ± SD; n = 3).**

The drug release profile is another important criterion while formulating polymeric nanoparticles. The profile of curcumin release in PBS (pH = 7.4) from the optimized formulation is illustrated in Figure 8. It was observed that the release consisted of an initial burst release phase corresponding to about 26% of drug release in the first hour, followed by a slow sustained release corresponding to 68% drug release in seven days and ~ 75% in 10 days. Sustained release kinetics where 75% curcumin was released from curcumin-PBCN nanoparticles over 24 h has been reported by Sun et al. [38]. In another study, Mohanty et al. (2010), showed 46% drug release in 24 h and 66% drug release over a period of 10 days from nanoparticlulate curcumin [39]. Release of curcumin from PLGA-CURC was more uniform and sustained over the 10 day period of study. The burst release of curcumin may be due to the surface associated curcumin bound weakly to the surface of the nanoparticles which gets released first. The remaining amount of curcumin which is encapsulated within the structure was released in a controlled manner for the entire period of study (10 days). Dissolution diffusion of the drug from the matrices and the slow matrix erosion are the mechanisms thought responsible for the slower drug release kinetics from the nanoparticles. 

Further, the release profile of curcumin from PLGA nanoparticles were investigated by using different release kinetic models: zero order, first order, Higuchi and Hixson-Crowell equations [26,27], and their regression coefficient (r^2^) was calculated from appropriate plots. The first order model describes the release to be concentration dependent while the Hixson– Crowell cube root model indicates a change in surface area or diameter due to erosion with progressive release of drug as a function of time. Release rate constants for burst release and sustained release are illustrated in Table 6. Comparing the amount of released curcumin with respect to time; for the burst release phase (first 6 h), PLGA-CURC followed the zero order model (R^2^ = 0.997). Higuchi kinetics model which states that diffusion is one of the major methods of drug release best described the controlled release phase (R^2^ = 0.996) during later part of the release which may be controlled by a combination of slow and gradual erosion and diffusion. Overall, the *in vitro* release data indicates that PLGA-CURC is capable of releasing curcumin in a controlled manner over a period of 10 days. 

**Table 6:** **Different kinetic models and regression coefficients of PLGA-CURC formulations**^**a**^

**Model**	**Equation**	**R**^**2**^**value for burst release (%)**	**R**^**2**^**value for sustained release (%)**
Zero order	$m_{0}-m=kt$	0.997	0.955
First order	lnm=kt	0.977	0.976
Higuchi’s model	$m_{0}-m=kt^{1/2}$	0.967	0.996
Hixson crowell	$m_{0}^{1/3}-m^{1/3}=kt$	0.987	0.955

^a^*m*_*0*_ is the initial drug amount (100%, when represented as percentage); m the amount of drug remaining at a specific time (calculated as % of *m*_*0*_); *k* the rate constant; *t* is the time.

#### Cellular uptake of nanoparticle prepared under optimal conditions

**Figure 9:**
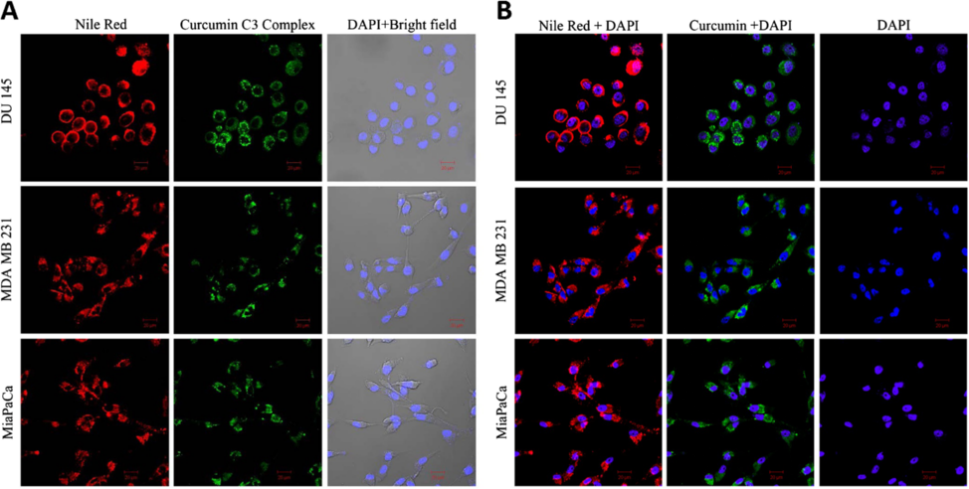
Confocal images of different cancer cell lines incubated with PLGA-CURC - (A) Red :- nile red-labeled PLGA-CURC, Green:- curcumin, Bright field merged with cell nuclei stained with DAPI; (B) Merged images of nile red-labeled PLGA-CURC with DAPI; Merged images of curcumin with DAPI and DAPI.

In order to study the uptake of PLGA-CURC by different cancer cell lines, we investigated the ability of nanoparticles to be endocytosed by the cells. Figure 9A and B illustrate panels of the confocal microscope images of different cancer cell lines incubated with Nile red-labeled PLGA-CURC for 2 h. Our results depict robust uptake of the nanoparticles in all the cell lines. The cells incubated with Nile red-labeled PLGA-CURC exhibited either red (due to Nile red) or green (due to curcumin) fluorescence, depending upon the excitation wavelength.

#### Western blot analysis

**Figure 10:**
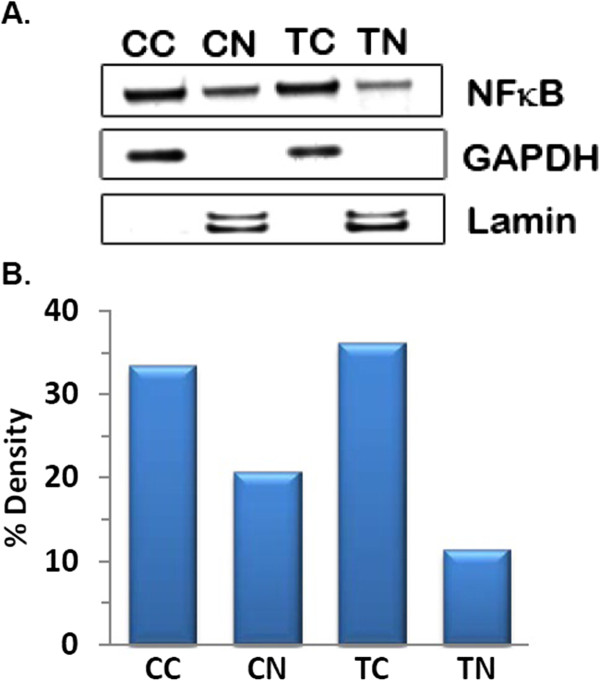
**A) Western blot showing inhibition of translocation of NFκB (p65) from cytosol to nucleus in MDA MB231 cells treated with PLGA-CURC.B**) Densiometric analysis of the western blot. (CC-Control cytosol extract, CN- Control nuclear extract, TC – Treated cytosol extract and TN – Treated nuclear extract).

A principal cellular target of curcumin in cancer cells is activated nuclear factor kappa B (NFκB) which is a family of five closely related proteins found in several dimeric combinations and bind to the NFκB consensus sequence on DNA [40]. NFκB is translocated to the nucleus from cytosol, where it induces the expression of more than 200 genes that have been shown to suppress apoptosis and induce cellular transformation, proliferation, invasion and metastasis. Many of these activated target genes are critical for establishment of the early and late stages of aggressive cancers. We studied the mechanism of action of PLGA-CURC on breast cancer cell line, MDA MB 231, and compared the functional pathways affected by PLGA-CURC to what has been previously reported for free curcumin [38]. The results of the Western blot analysis, as seen in Figure 10, depicts that PLGA-CURC was able to inhibit the translocation of NFκB from cytosol to nucleus in MDA MB-231 cells. The degree of inhibition with PLGA-CURC treatment was seen to be greater as compared to untreated cells, as depicted with fainter bands of NFκB corresponding to the nuclear extract from cells treated by PLGA-CURC. This result illustrates that the curcumin encapsulated within the PLGA-CURC retains its functional activity on encapsulation and subsequent release from the nanoparticles. 

#### Pharmacokinetic Studies

**Figure 11:**
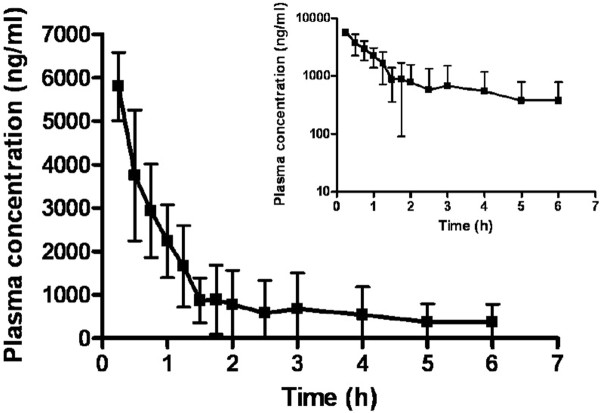
**In vivo bioavailability of curcumin using PLGA-CURC nanoparticles.** PLGA-CURC were administered intravenously to the rats at a dose of 7.5 mg/kg (n = 6).

PLGA-CURC were formulated to improve the bioavailability of curcumin. To evaluate this, male Sprague Dawley rats were administered PLGA-CURC nanoparticles intravenously (7.5 mg/kg equivalent curcumin nanoparticles). Blood samples were collected at predetermined time intervals and the concentration of curcumin was determined by HPLC analysis. Our results showed (Figure 11) that the pharmacokinetic profile of PLGA-CURC in rats by i.v. administration followed two compartmental model. The area under the curve (AUC_0-∞_) after i.v. injection of PLGA-CURC was found to be 6.139 mg/L h. This value is much higher compared to 3.16 mg/L h reported by Ma et al. (2007) for their micellar formulation of curcumin at a dose of 5 mg/kg [41]. Duan et al. (2010) reported the area under the curve to be 3.302 mg/L h for a 5 mg/kg dose of their curcumin-PBCA nanoparticles. Both these groups reported AUC_0-∞_ of free curcumin at 10 mg/kg dose to be only 1.67 mg/L h and 1.92 mg/L h respectively [42]. They also reported a higher elimination half life of curcumin when encapsulated within nanoparticles or micelles alongwith a decrease in clearance rate. Our pharmacokinetic results for PLGA-CURC showed the same trend. This is expected when the drug in circulation is restricted to the blood compartment because of being encapsulated with nanoparticles [42]. The higher level of curcumin concentration observed in the case of PLGA-CURC nanoparticles might be explained by increased bioavailability as a function of increased aqueous dispersibility, smaller nanoparticle size and increased accumulation of nanoparticles in different organs together with sustained release of curcumin from them. Similar observations related to pharmacokinetic studies of curcumin or nanoparticles have been reported by various other groups [9,38,42]. 

#### Storage stability of PLGA-CURC nanoparticles

**Figure 12:**
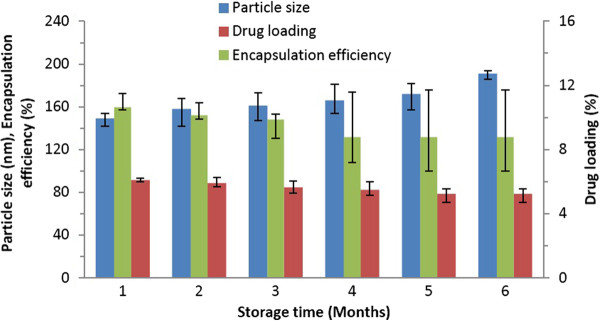
The particle size, encapsulation efficiency and drug loading of PLGA-CURC against storage time at 4°C.

The long term storage stability of the PLGA-CURC is an important parameter when scaling up the formulation. Nanoparticle formulations increase the surface area by many folds and this may lead to very high aggregation after long periods of storage. This poor long term stability may be due to different physical and chemical factors that may destabilize the system [43]. Lyophilization is a promising approach for the stabilization of PLGA nanoparticles [44]. For lyophilized nanoparticles, cryoprotectants serve as stabilizers during the freeze drying process. For our study, sucrose (5% w/v) and trehalose (2% w/v) were chosen as the cryoprotectants to prevent the hydrolytic instability, aggregation between nanoparticles, protection during processing and storage. After 6 months of storage with cryoprotectants at 4°C, the nanoparticles appear to be stable without any collapse or aggregation. Figure 12 shows effect of long term storage on the particle size, encapsulation efficiency and drug loading of nanoparticles. We saw no major changes besides a slight increase in particle size and a slight decrease in encapsulation efficiency and drug loading. Therefore, PLGA-CURC formulated by our s-o/w emulsion solvent evaporation and diffusion technique was found to be stable for a long period of time. 

#### Gamma irradiation PLGA-CURC nanoparticles

**Figure 13:**
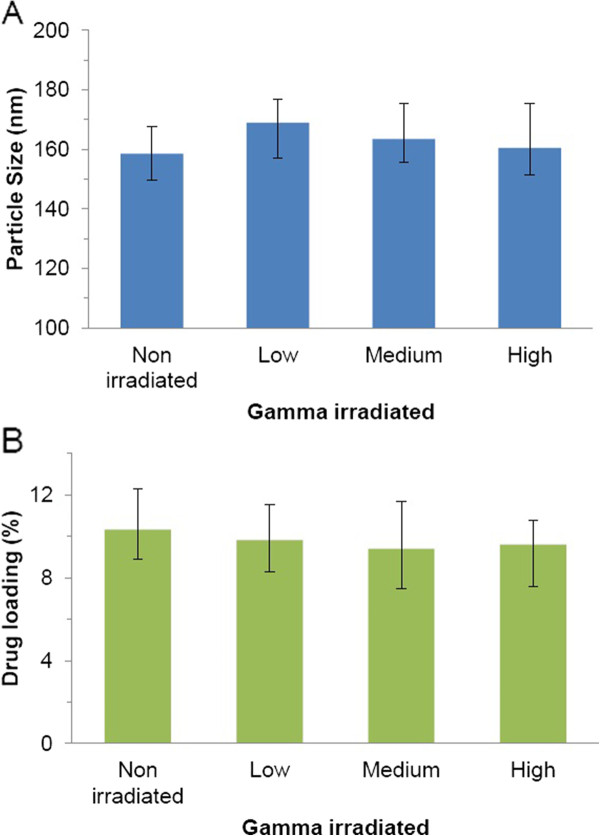
The particle size (A) and drug loading (B) of PLGA-CURC after γ-irradiation at doses 16.8 kGy for 241 minutes (Low), 25.3 kGy for 179 minutes (Medium) or 35.8 kGy for 241 minutes (High) (n = 3).

Gamma irradiation is critical as it renders sterility to the nanoparticles before being injected into the body [45]. There are many alternative techniques for sterilization but we chose γ-irradiation as it is known for its high penetration power and isothermal property of gamma rays that permits sterilization of even sensitive materials [46]. However, γ-irradiation may have some effects on the nanoparticle size or drug loading. The changes in particle size and drug loading for low, medium and high exposures are graphed in Figure 13. Our results demonstrate that there were no statistically different changes observed between non-irradiated and different dose irradiated nanoparticles. Further, γ-irradiation did not alter the drug loading in the nanoparticles. 

## Conclusion

Scaling up of the nanoformulation process is essential for the future development of nanoparticle based drug delivery technologies. In this paper, we have made a successful effort towards formulating, optimizing and scaling up PLGA-CURC by using Solid-Oil/Water emulsion technique. Once formulation was achieved, we optimized our process by successful use of RSM using CCD model and scaled up the formulational process in four stages with final scale-up process yielding 5 g of PLGA-CURC. The major goals while designing the scale up stages were to control particle size and polydispersity while maximizing drug encapsulation efficiency and drug loading which were adequately achieved. PLGA-CURC, under the optimized conditions were found to have a particle size of 158.5 ± 9.8 nm, polydispersity of 0.141 ± 0.011, encapsulation efficiency of 90.34 ± 2.3% and drug loading of 10.32 ± 1.4%. Morphological studies of the final scaled up batch showed that the PLGA-CURC were smooth, spherical with a uniform surface. The release kinetics from PLGA-CURC exhibited a biphasic pattern with an initial burst release followed by a slower diffusion controlled drug release for a period of 10 days. Intracellular uptake studies revealed excellent uptake in prostate, breast and pancreatic cell lines. Pharmacokinetic studies illustrated AUC_0-∞_ of PLGA-CURC after i.v. injection to be 6.139 mg/L h which is higher than most reported in literature for curcumin based formulation. Stability analysis showed long term physicochemical stability and gamma irradiation studies showed no significant changes after sterilization of the PLGA-CURC formulation. In conclusion, our nanoformulation, PLGA-CURC, significantly overcame the limitation of the lack of aqueous solubility of curcumin and thereby improved its bioavailability. The formulation process was successfully optimized using CCD-RSM and scaled up to produce 5 g of PLGA-CURC with similar physicochemical characteristics as that of the primary formulated batch. This scale-up process can be further elaborated to produce higher quantities which would prove beneficial for efficient manufacturing at an industrial scale.

## Competing interests

This work was performed under a sponsored research agreement between SignPath Pharma Inc. and UNTHSC. Conceptualization, design and performance of experiments were by AR, AM and JKV. This product technology has been licensed by SignPath Pharma Inc. from UNTHSC.

## Authors’ contributions

APR and AM performed all optimization experiments, analyzed data and wrote the manuscript. JKV supervised and mentored work and corrected the manuscript. LH provided the funds in part for this research and reviewed the manuscript. All authors read and approved the final manuscript.

## Supplementary Material

Additional file 1 Table S1 The analysis of variance for the response surfaces obtained for particle size, polydispersity, encapsulation efficiency and drug loading. (PDF 16 kb) Click here for file
