# Supplementary material for: Scale up, optimization and stability analysis of Curcumin C3 complex-loaded nanoparticles for cancer therapy
Source: J Nanobiotechnology. 2012 Aug 31;10:38. doi: 10.1186/1477-3155-10-38 (PMC3497871; doi:10.1186/1477-3155-10-38)
Supplement: Additional file 1 Table S1 — The analysis of variance for the response surfaces obtained for particle size, polydispersity, encapsulation efficiency and drug loading. (PDF 16 kb) [file 1477-3155-10-38-S1.pdf]

Supplement pages

Table 1: The analysis of variance for the response surfaces obtained for particle size, polydispersity, encapsulation efficiency and drug loading.

Measure	Particle size	Polydispersity	Encapsulation efficiency	Drug loading
**F value**	30.87	9.25	14.15	9.37
**P>F**[a]	<0.0001	0.0009	0.0001	0.0008
**Degree of freedom of model**	9	9	9	9
**RMSE**	7.36	0.012	4.79	1.05
**R^2**	0.9653	0.8927	0.9273	0.8940
**Coefficienct of variance**	4.85	8.93	6.10	7.73
**Adequate precision**[b]	24.433	12.691	10.920	13.445

[a] Values indicate that confidence level for particle size polydispersibility, encapsulation efficiency and drug loading are >99.9 %.

[b] Value >4 indicates adequate precision in the model
